# Pre- and In-Hospital Non-Invasive Ventilation

**Published:** 2011-10-17

**Authors:** E. De Robertis, M. Iannuzzi, R. Tufano, O. Piazza

**Affiliations:** Dipartimento di Scienze Chirurgiche Anestesiologiche Rianimatorie e dell’Emergenza, Università degli Studi di Napoli “Federico II”; § Facoltà di Medicina e Chirurgia, Università degli Studi di Salerno

The rational of mechanical ventilation is to support gas exchange and unload respiratory muscle until the pathophysiology leading to respiratory failure improves. Intubation maintains patent airways, allows high working pressure but significantly increases the risk to develop severe respiratory infections and is poorly tolerated by the patients.

Over the last two decades devices have been produced to consent a non-invasive interface between patient and ventilator. Today it is thus possible to ventilate patients with highly performing and well tolerated face masks of different shapes or with helmets. Moreover, ventilators have been implemented with modalities of ventilation aimed at a non-invasive ventilation (NIV).

In NIV several ventilator modalities can be used. The most used are: continuous positive airway pressure (CPAP) that delivers constant pressure during both inspiration and expiration, and that does not actively assist inspiration; and differently delivered forms of bi-level ventilation (NiPPV) which provide two levels of airway pressure that actively assist inspiration while providing end expiratory pressure.

These implementations have challenged the way NIV can be used and have open new approaches. Today NIV can be used to prevent intubation, as an alternative to conventional invasive ventilation, or to prevent re-intubation after weaning.

It is not surprising that citations in PubMed of articles addressing NIV have been increased over the last two decades, together with a widespread use of NIV in several pathologies as a proportion of invasive mechanical ventilation **(1)**.

Today, among intensivists patients suffering from exacerbation of chronic obstructive pulmonary diseases (COPD), cardio-pulmonary edema (CPE), neuromuscular diseases, obesity and others pathologies are mainly ventilated with a NIV approach. However, in emergency department, although the perception of the utility of NIV is high, few patients benefit today of a NIV treatment **(1, 2)**.

An early NIV approach may have good reasons but can rises many criticisms. We will shortly discuss the rational for an early NIV approach and propose organizational solutions to offer to a large population of patients NIV in a safe way under the management of expert specialist such as anesthetists or intensivists.

First of all we should briefly discuss which pathologies can benefit more of NIV.

Many articles have evaluated NIV in patients suffering from CPE. A recent meta-analysis evaluating CPAP and NiPPV in CPE has evidenced that in this population both CPAP and NiPPV, compared with a standard therapy, significantly reduce the need for subsequent mechanical ventilation and mortality; NIPPV seems to give only slightly better results over CPAP **(3)**. Accordingly to these results NIV seems to be a safe approach in CPE.

Patients suffering from acute respiratory failure (ARF) are more heterogeneous and it is necessary to separately evaluate the effects of NIV in hypoxic ARF and in hypercapnic ARF, due to exacerbation of COPD.

As concern NIV in COPD, more than 17 trials have been published. At the list 4 trials have shown a significantly better outcome for the patients treated with NIV compared with standard therapy. The other trials have outlined a significant effect of NIV on symptomatology with better blood gases exchange, reduction in heart rate and respiratory rate. **(4)**.

Only 4 trials have evaluated NIV in patients suffering from hypoxic ARF and all have failed to show a benefit of NIV in this population **(4)**. However, different results have been observed when NIV was evaluated in subgroups of hypoxic ARF. Authors have shown significant effects of NIV, compared with standard treatment, on need for mechanical ventilation and mortality in patients suffering from pneumonia **(5, 6)** or immunocompromised **(7).**

The scant effect observed in hypoxic ARF rises some doubts and many questions: how important is to offer as early as possible a positive pressure ? Can an early positive pressure positively interfere with the progression of the pathology ? Is it possible that trials in hypoxic ARF are biased by a too late NIV support in the progression of ARF ?

Since the first description of ARDS, it is known the importance of early positive pressure for the treatment of these patients **(8)**. Time factor is of a crucial importance and NIV offer the possibility to offer a positive pressure in a very early stage of ARF, when intubation would be unthinkable.

In the recent Virus A H_1_N_1_ outbreaks of 2009–2011 we have, contrary to most of the literature, used an early NIV approach in severely ARF hypoxic patients. Our preliminary data show that all patients treated with NIV had a good outcome. Patients traditionally ventilated in which positive pressure was started later in the pathology development did worst. From our preliminary analysis it seems that time elapsed from the start of flu symptoms to positive pressure is an independent factor of mortality.

But which evidences do we have of the efficacy and safety of an early NIV approach ? Up to now no trials with the power of offering us an answer have been published but numerous papers offer us the flavor of a better outcome of patients treated with early NIV.

Thompson et al., in a randomized controlled trial enrolling 71 patients, mainly COPD and CPE, showed that a pre-hospital early NIV approach decreased by 30% the need of intubation and reduced by 21 % mortality. It is important to point out the fact that the authors to emphasize the results enrolled only severe respiratory failure at risk of intubation **(9)**.

A recent meta-analysis has evidenced that early pre-hospital use of NIV in CPE significantly improve short term mortality, decreases the need for intubation and improves acute symptomatology. From this meta-analysis remains unclear if benefits of early pre-hospital NIV in relation to patient outcome are considerable enough to justify the significant organisational cost associated **(10)**.

The economic burden of a widespread pre-hospital NIV use is particularly important and should be considered in the light of the few emergency vehicles staffed with devices for NIV. It has been evidenced that in three well developed central Europe countries also helicopters are generally not staffed with ventilators able to deliver NIV, masks and helmets **(11)**.

If we now analyze the in-hospital use of NIV many authors have evidenced the utility of early NIV in COPD patients in terms of reduced need for intubation and control of symptomatology **(12)**.

Recently an early in-hospital NIV use in CPE has been evaluated in a randomized trial performed in emergency departments in UK. After the randomization of 1069 patients, a group of patients received standard care, a group was treated with CPAP and a third group with NiPPV. Non-invasive ventilatory support delivered by either CPAP or NIPPV safely provided earlier improvement and resolution of breathlessness, respiratory distress and metabolic abnormality. However, this was not translated into improved shorter longer-term survival. The authors concluded recommending that CPAP or NIPPV should be considered as adjunctive therapy in patients with severe acute cardiogenic pulmonary edema in the presence of severe respiratory distress or when there is a failure to improve with pharmacological therapy. The authors also evaluated the costs of NIV use and concluded that CPAP should be the non-invasive ventilation modality of choice, as NIPPV provides no additional benefit over CPAP and CPAP equipment is less complex and less expensive **(13)**.

Recently, the differences in CPE patients of CPAP and NIPPV have been re-evaluated in a multicenter randomized study that assigned 200 patients to receive CPAP or NIPPV. The authors concluded that during CPE, NIPSV accelerates the improvement of respiratory failure compared to CPAP but does not affect primary clinical outcome either in overall population or in subgroups of patients with hypercapnia or those with high B-type natriuretic peptide **(14)**

These results have well known physiological basis. It has been clearly evidenced that CPAP improves oxygenation but fails to unload the respiratory muscles, while pressure support + PEEP is needed to reduce inspiratory muscle effort and dyspnoea **(15).**

In a recent editorial L’Her has pointed out that in an ICU setting, NIPPV would probably be the best choice; the use of cheap, and simple CPAP devices during the pre-hospital care could be considered a better one, especially due to volumetry constraint and to the low efficiency of pressure support modes from several emergency and transport ventilators. On a routine basis, beside real differences in terms of physiological improvements between CPAP and NIPPV, the choice of one ventilatory mode over another is based primarily on the physicians’ expertise and the operation setting **(16).**

But if an early NIV approach is effective and we enlarge the number of patients ventilated with NIV, two problems immediately do rise: the bottle neck of the few ICUs beds and all safety aspects of a NIV performed in environments not prepared and not adequately staffed to face a patient rapid deterioration.

In a recent Italian survey it has been observed that NIV is often performed outside ICUs in emergency departments and/or general wards. However, personnel training preceding NIV was not widespread and protocols were not always present. Moreover, the efficacy of NIV was perceived low, monitoring was usually limited, reported complications and practical problems were potentially severe. The authors concluded pointing out that although NIV is extensively applied in Italy in non-ICU environments, many criticalities and contradictions have not been solved yet **(17)**.

From this experience it is clear that solutions should be proposed for a widespread NIV use.

A Japanese group has proposed a quick and broad application of NIV in the emergency department and continue it in an intermediate-care-unit for every possible non contraindicated patient with acute respiratory failure needing ventilator support. The authors have observed an overall significant reduction in in-hospital mortality and use of the ICU and intermediate-care-unit in emergent admissions of patients with ARF of various pulmonary etiologies. The immediate transfer of patients to an intermediate-care-unit has in fact limited ICU use for the worst situations. The authors have thus safely enlarged the population of patient that could benefit of NIV, reducing at the same time the use of ICU. **(18)**.

Recently, in our opinion, a more appropriate solution has been proposed: NIV managed outside intensive care units by anaesthesiologists acting as a medical emergency team. The reported overall success rate was 77.5%, 10.1% of the patients were intubated and 12.4% died (all of them were “do not attempt resuscitation” patients). Complications were limited, the work-load for the medical emergency team sustainable. The authors concluded that under the supervision of a medical emergency team, NIV could be applied in a wide variety of settings, outside the ICU, with a high success rate and with few complications. **(19)**.

NIV requires an appropriate environment with the continuous presence of adequate staff with expertise in NIV, monitoring facilities, rapid access to IOT and invasive ventilation. Obviously, ICU setting fits all these criteria.

The model of hospital care differs from country to country and the type of treatment undertaken in a given department differs from hospital to hospital. Issues of staffing and training in a general ward where nurse to patient ratio is commonly modest, enlarging the capabilities of intermediate-care-unit or referring to experienced specialists to supervise NIV remains in the decisions of each country and each Institution.

We believe and strongly support the idea that initiation and continuation of NIV outside ICUs under the supervision of a medical emergency team staffed by anaesthesiologists expert in non-invasive ventilation is an attracting safe and less expensive alternative. Today the use of wireless technology allows us to control a great number of patients at distance in the hospital. Creating team devoted to NIV allow to offer our deep-rooted expertise in ventilation to a wide population exporting it outside ICUs.
